# Increased expression of pathological markers in Parkinson’s disease dementia post-mortem brains compared to dementia with Lewy bodies

**DOI:** 10.1186/s12868-021-00687-4

**Published:** 2022-01-04

**Authors:** Haitao Tu, Zhi Wei Zhang, Lifeng Qiu, Yuning Lin, Mei Jiang, Sook-Yoong Chia, Yanfei Wei, Adeline S. L. Ng, Richard Reynolds, Eng-King Tan, Li Zeng

**Affiliations:** 1 Neural Stem Cell Research Lab, Research Department, National Neuroscience Institute, Singapore, 308433 Singapore; 2 Guangxi University of Chinese Medicine, 179 Mingxiu Dong Rd., Nanning, 530001 Guangxi China; 3 Department of Anatomy and Neurobiology, Zhongshan School of Medicine, Sun Yat-Sen University, #74, Zhongshan No. 2 Road, Guangzhou, 510080 China; 4 Department of Neurology, National Neuroscience Institute, Singapore, 308433 Singapore; 5 DUKE-NUS Graduate Medical School, Neuroscience & Behavioral Disorders Program, Singapore, 169857 Singapore; 6 Division of Neuroscience, Imperial College London, Hammersmith Hospital, London, W12 0NN UK; 7 Centre for Molecular Neuropathology, Lee Kong Chian School of Medicine, Nanyang Technological University, Novena Campus, 11 Mandalay Road, Singapore, 308232 Singapore; 8 Department of Human Anatomy, Institute of Stem Cell and Regenerative Medicine, Dongguan Campus, Guangdong Medical University, Dongguan, China

**Keywords:** Parkinson’s disease dementia (PDD), Dementia with Lewy bodies (DLB), Alzheimer’s disease pathogenesis, Biomarkers, Post-mortem brain tissue

## Abstract

**Background:**

Parkinson’s disease (PD) and dementia with Lewy bodies (DLB) are common age-related neurodegenerative diseases comprising Lewy body spectrum disorders associated with cortical and subcortical Lewy body pathology. Over 30% of PD patients develop PD dementia (PDD), which describes dementia arising in the context of established idiopathic PD. Furthermore, Lewy bodies frequently accompany the amyloid plaque and neurofibrillary tangle pathology of Alzheimer’s disease (AD), where they are observed in the amygdala of approximately 60% of sporadic and familial AD. While PDD and DLB share similar pathological substrates, they differ in the temporal onset of motor and cognitive symptoms; however, protein markers to distinguish them are still lacking.

**Methods:**

Here, we systematically studied a series of AD and PD pathogenesis markers, as well as mitochondria, mitophagy, and neuroinflammation-related indicators, in the substantia nigra (SN), temporal cortex (TC), and caudate and putamen (CP) regions of human post-mortem brain samples from individuals with PDD and DLB and condition-matched controls.

**Results:**

We found that p-APP^T668^ (TC), α-synuclein (CP), and LC3II (CP) are all increased while the tyrosine hydroxylase (TH) (CP) is decreased in both PDD and DLB compared to control. Also, the levels of Aβ42 and DD2R, IBA1, and p-LRRK2^S935^ are all elevated in PDD compared to control. Interestingly, protein levels of p-Tau^S199/202^ in CP and DD2R, DRP1, and VPS35 in TC are all increased in PDD compared to DLB.

**Conclusions:**

Together, our comprehensive and systematic study identified a set of signature proteins that will help to understand the pathology and etiology of PDD and DLB at the molecular level.

**Supplementary Information:**

The online version contains supplementary material available at 10.1186/s12868-021-00687-4.

## Background

Parkinson’s disease (PD) is a chronic neurodegenerative movement disorder characterized clinically by limb rigidity, bradykinesia, tremor, and postural instability. Approximately 30% of PD patients have mild cognitive symptoms at the time of motor symptom onset, and approximately 80% will develop Parkinson’s disease dementia (PDD) during the course of the disease [1, 2]. Dementia with Lewy bodies (DLB) is characterized by the deposition of Lewy bodies, which contain abnormally folded α-synuclein in neurons. Patients with DLB present with prominent early cognitive impairment, visual hallucinations, REM sleep behavioral disorders, and fluctuating attention/cognition, followed by parkinsonism [3, 4]. The prevalence of DLB may be as high as 26% of subjects in individual clinical or community-based cohorts of Parkinsonism [5]. Together, PDD and DLB comprise Lewy body dementia (LBD) spectrum disorders associated with cortical and subcortical Lewy body pathology.

Currently, patients with PDD and DLB are distinguished clinically by their neuropsychological profiles and clinical presentations [3, 6]. According to the criteria set by the Lewy Body Consortium [4], DLB is defined by an earlier onset of dementia after movement problems, i.e., less than one year after the onset of motor symptoms, while PDD is characterized by a later onset of dementia after movement problems, i.e., at least 1 year after the onset of motor symptoms, typically 10–15 years after initial PD diagnosis [1, 7]. Their similar overlapping clinical-pathological profiles frequently confound clinical diagnosis, and a variety of clinical and neuropsychological measures have been applied to aid in diagnosis [4, 8].

In addition to neuropsychological and clinical characterizations, efforts have been devoted to the development of biomarkers for differentiating PDD and DLB, which is important for diagnosis. Synaptic protein loss is a common pathological feature in dementia, and synaptic changes may occur before neurodegeneration [9]. Alpha-synuclein has been reported to be involved in the changes that occur in synaptic proteins [10], a common feature in synucleinopathies including PDD and DLB [11, 12]. Recently, using an in-depth proteomic approach, Bereczki et al*.* identified a series of synaptic biomarkers, such as the presynaptic proteins SNAP47, Rab3A, GAP43, and SYBU, which were altered in cerebrospinal fluid (CSF) and brain samples in patients with PDD, DLB and Alzheimer’s disease (AD) [12, 13]. However, these studies did not test other proteins that are important in PDD and DLB pathogenesis such as mitophagy proteins, mitochondrial dynamic proteins, and neuroinflammation markers. Furthermore, CSF protein levels may not directly or accurately reflect the levels within the brain. In 2017, Zhao et al. measured LRRK2, p-LRRK2^S935^, VPS35, GBA, MPR300, and IGF2R levels in post-mortem brain samples from PD patients carrying LRRK2 mutations to study the relationship between LRRK2 and retromer dysfunction in LRRK2-associated PD [14]. They only used brain tissue from the frontal cortex of 17 patients with LRRK2-associated PD, without comparing samples from DLB patients. In the search for biomarkers to differentiate PDD from DLB, a more systematic and holistic approach is needed.

Mitochondria are important in many biological processes, including cell respiration, metabolism, energy production, oxidative stress, and apoptosis [15, 16]. Mitochondrial dysfunction is present in more than 50 diseases, including cancer and neurodegenerative diseases such as AD and PD [17, 18]. Aβ accumulation and the hyperphosphorylation of Tau can lead to the dysfunction of mitochondria in AD [19]. Aβ-induced toxicity results in the disruption of mitochondrial DNA maintenance, electron transport chain, and protein import machinery [20, 21]. The aggregation of insoluble α-synuclein containing Lewy bodies is the classic pathological hallmark of PD. Studies show that α-synuclein can negatively affect mitochondrial function and dynamics [22]. Mitophagy is the selective removal of dysfunctional mitochondria by autophagy and is a key quality control step in maintaining the homeostasis and integrity of the mitochondrial network [23]. The dysregulation of mitophagy contributes to the onset and progression of neurodegenerative diseases, including AD and PD [24].

To systematically study AD- and PD-associated proteins in PDD and DLB, we examined human post-mortem brain samples. A total of 28 cases were studied, comprising those from nine controls, 10 patients with PDD, and nine patients with DLB, each case contains the temporal cortex (TC), substantial nigra (SN), and caudate and putamen (CP). We found that expression levels of AD-associated protein Aβ42 were increased in PDD samples than in controls in the TC region. Levels of PD-associated proteins tyrosine hydroxylase (TH) were reduced in PDD and DLB as compared to control, whereas α-synuclein was increased in DLB than control in the CP region. Also, the neuroinflammation protein IBA1 in the TC region and the autophagy proteins LC3II in the CP region were elevated in both PDD and DLB when compared to the control. Moreover, LRRK2 substrates Rab10 and p-LRRK2^S935^ were increased in the TC region of PDD than in the control. Notably, levels of Aβ42, DD2R, DRP1, p-LRRK2^S935^, and VPS35 in the TC region and p-Tau^S199/202^ in the CP are higher in PDD than in DLB. The protein signatures identified in our systematic study will help to better understand the pathology and etiology of PDD and DLB at the molecular level.

## Results

### Demographic characteristics of the post-mortem brain tissue samples

To identify signature protein markers in PDD and DLB, post-mortem brain tissues were examined in nine age-matched controls, 10 PDD, and nine DLB cases. Subjects with non-neurological or low clinical AD presentation were considered as controls for PDD and DLB. Each case contains samples from the temporal cortex (TC), substantial nigra (SN), and caudate and putamen (CP) region. The detailed demographic characteristics of the individuals that provided the samples are listed in Table 1. The cause of death, clinical diagnosis, and pathological feature of each case were carefully examined by their clinical doctors and doctors from the UK Human Tissue Authority. According to the clinical diagnosis, including disease history, clinical presentation, dementia scores, alpha-synuclein pathological feature, and phosphor-Tau- and Aβ-related pathological features, the cases were classified as the control, PDD, and DLB patients by their clinical doctors (Additional file 1: Table S1), which were used as the three study groups investigated here. The sample sizes, ages at death, and gender ratios were balanced among the groups, and there were no significant differences between the control, PDD, and DLB groups. In Table 1, we found that the onset age of PDD is slightly earlier than that of DLB. Notably, the symptom duration in PDD patients was significantly longer than that of DLB patients (16.8 ± 2.7 and 6.1 ± 1.3 years, respectively, *p*  = 0.0032) (Table 1). This is consistent with previous studies showing that PDD has an earlier onset and with longer duration [1, 7]. In DLB patients, early motor symptoms associated with PDD were not prominent, hence it may take a longer time to develop and diagnose.

**Table 1:** Demographic details of samples used in this study

	Control	PDD	DLB
Sample size	9 (TC)	10 (TC)	9 (TC)
	9 (SN)	10 (SN)	9 (SN)
	9 (CP)	10 (CP)	9 (CP)
Gender (male/female)	6/3	6/4	6/3
Post-mortem interval, hours	19.8 ± 1.5 (12–25)	14.1 ± 1.9 (2–22)	15.6 ± 2.0 (6–24)
Onset age (years)	NA	63.1 ± 3.7 (42–80)	67.8 ± 3.9 (43–80)
Symptom duration (years)	NA	16.8 ± 2.7 (5–34)	6.1 ± 1.3** (1–15)
Dementia age (years)	NA	76.5 ± 1.7 (65–82)	69.7 ± 3.1 (54–81)
Age at death (years)	83.2 ± 3.6 (66–98)	79.7 ± 1.2 (75–85)	73.7 ± 2.8 (58–83)

Demographic data for the human post-mortem brain tissue cases in this study are shown. Kruskal–Wallis test was used to measure differences among Control, PDD, and DLB for post-mortem interval and age at death. A two-tailed student’s *t *test with equal variance was used to analyze statistical differences between PDD and DLB for onset age, symptom duration, and dementia age. Data are presented as the mean  ±  SEM and the ranges are shown in parentheses

*TC *temporal cortex; *SN *substantia nigra; *CP *caudate and putamen; *NA *not applicable

**p  < 0.01

### Expression of AD pathogenesis markers Aβ42 and p-APP^T668^ are increased in PDD in TC

**Fig. 1:**
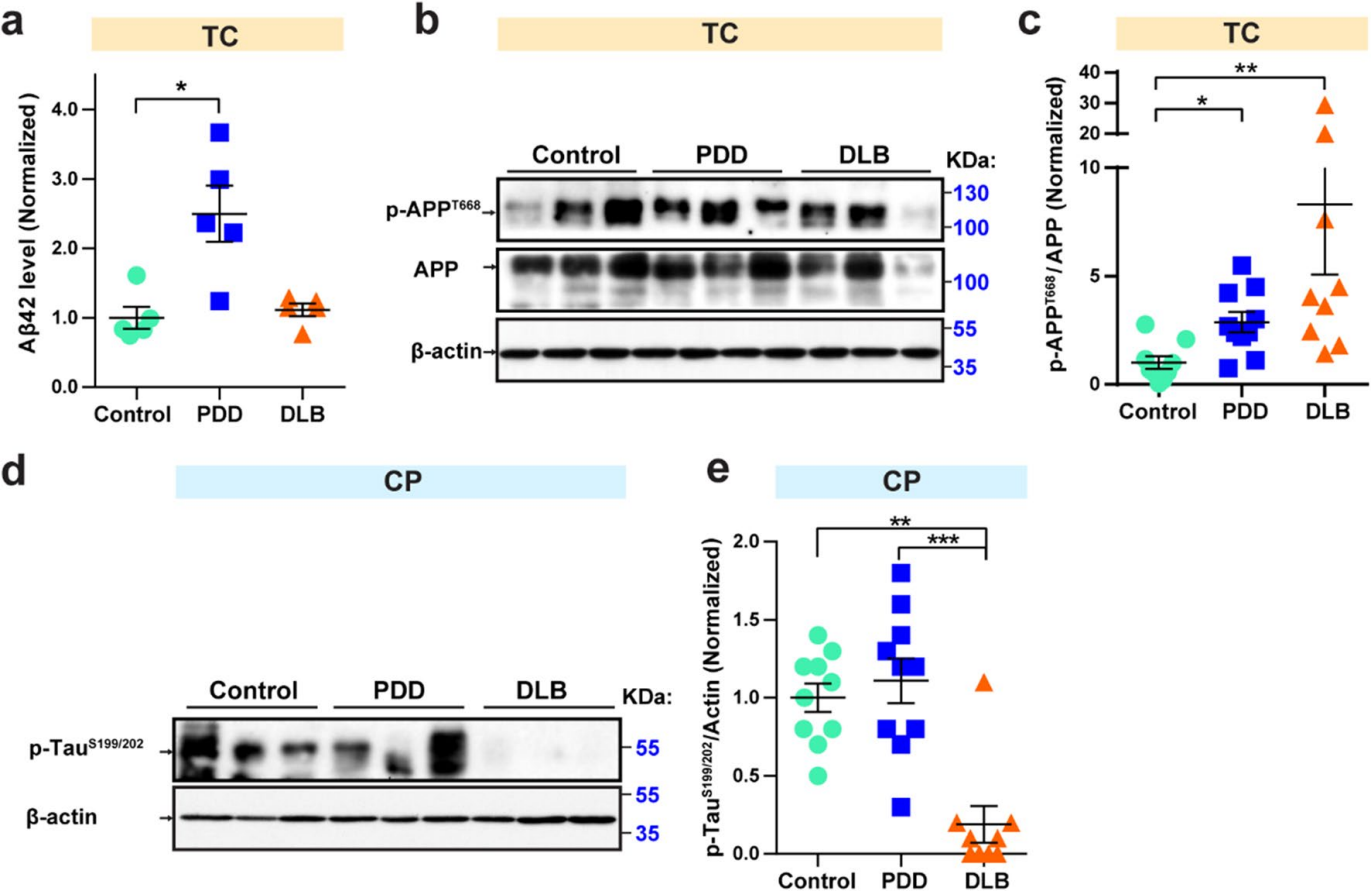
Increased Aβ42 in PDD and decreased p-Tau^S199/202^ levels in DLB. Multivariate analysis of post-mortem samples from the TC and CP regions of controls and individuals with PDD and DLB. **a** Protein levels of Aβ42 in the TC by ELISA assay; 100 µg of each protein sample was used per ELISA reaction. N  = 5. **b** p-APP^T668^ and APP protein levels were measured in the TC samples by western blot. Twenty micrograms of protein were used per well for SDS-PAGE. Protein values are normalized to β-actin levels and control samples. Blots were cropped from different gels. Representative western blot images were shown. All blot images with significant changes were included in the Additional file 1: Fig. S1. **c** Statistical results of relative p-APP^T668^ protein levels normalized to APP levels in the TC. **d** p-Tau^S199/202^ protein levels were measured in CP by western blot. Blots were cropped from different gels. Representative western blot images were shown. Protein values are normalized to β-actin levels and control samples. **e** Statistical results of relative p-Tau^S199/202^ levels in the CP samples. Data are presented as the mean  ±  SEM. Kruskal–Wallis test was used to measure the differences between Control, PDD, and DLB samples. *p  < 0.05, **p  < 0.01, ***p  < 0.001. Sample numbers, Control  = 9, PDD  = 10, and DLB  = 9

Dementia, which is the hallmark of AD, is also a common feature of PDD and DLB. We therefore first examined the protein levels of AD pathogenic markers, including Aβ42 and the phosphorylation of Tau at Ser-199 and Ser-202 (p-Tau^S199/202^) and phosphorylation of APP at Thr-668 (p-APP^T668^) in post-mortem samples. Aβ42 protein levels in the TC region were detected by the ELISA assay. In the TC region, compared to control samples, we found that Aβ42 protein levels were increased 1.5-fold in PDD samples (*p*  = 0.022) (Fig. 1a). Notably, when normalized to total APP, p-APP^T668^ protein levels were increased 1.9-fold (*p*  = 0.035) in PDD and 7.3-fold (*p*  = 0.002) in DLB samples compared to control samples (Fig. 1b, c). In the CP region, compared to control samples, the p-Tau^S199/202^ levels were significantly decreased by 81.1% (*p*  = 0.007) in DLB samples (Fig. 1d, e). Compared to DLB samples, p-Tau^S199/202^ protein levels were significantly increased 4.9-fold (*p * = 0.001) in PDD (Fig. 1d, e). We found that there were no significant (NS) difference  in the AD-related protein markers that we tested in the SN region of control, PDD, and DLB (Table 2). Taken together, our data demonstrate that level of AD pathogenic marker of Aβ42 is increased in PDD and the relative p-APP^T668^ protein levels are enhanced in both PDD and DLB in the TC region. In the CP region, p-Tau^S199/202^ protein levels are significantly decreased in DLB compared to control and increased in PDD compared to DLB.

**Table 2:** Statistic results of AD-related protein markers tested in three regions of Control (Ctrl), PDD, and DLB post-mortem samples

Protein	Region	PDD/Ctrl	DLB/Ctrl	PDD/DLB
Aβ-42	TC	↑* (0.022)	NS	NS
p-Tau^S199/202^	SN	NS	NS	NS
	CP	NS	↓** (0.007)	↑*** (0.001)
	TC	NS	NS	NS
APP	SN	NS	NS	NS
	CP	NS	NS	NS
	TC	NS	NS	↑* (0.016)
p-APP^T668^	SN	NS	NS	NS
	CP	NS	↑* (0.038)	NS
	TC	↑** (0.006)	↑* (0.021)	NS
p-APP^T668^/APP	SN	NS	NS	NS
	CP	NS	NS	NS
	TC	↑* (0.035)	↑** (0.002)	NS

Statistical results from western blot results for AD-related protein markers tested in this study. Western blot images were quantified using ImageJ, and the data were normalized to β-actin. The upwards arrow indicates an increase and the downwards arrow indicates a decrease. *p*  < 0.1 was included in the bracket to show the trends of the protein levels. Kruskal–Wallis test was used to measure the differences between Control, PDD, and DLB samples

*NS *no significance

*p  < 0.05, **p  < 0.01, ***p  < 0.001

### Expression of PD pathogenesis marker TH is decreased and α-synuclein is increased in both PDD and DLB in CP

**Fig. 2:**
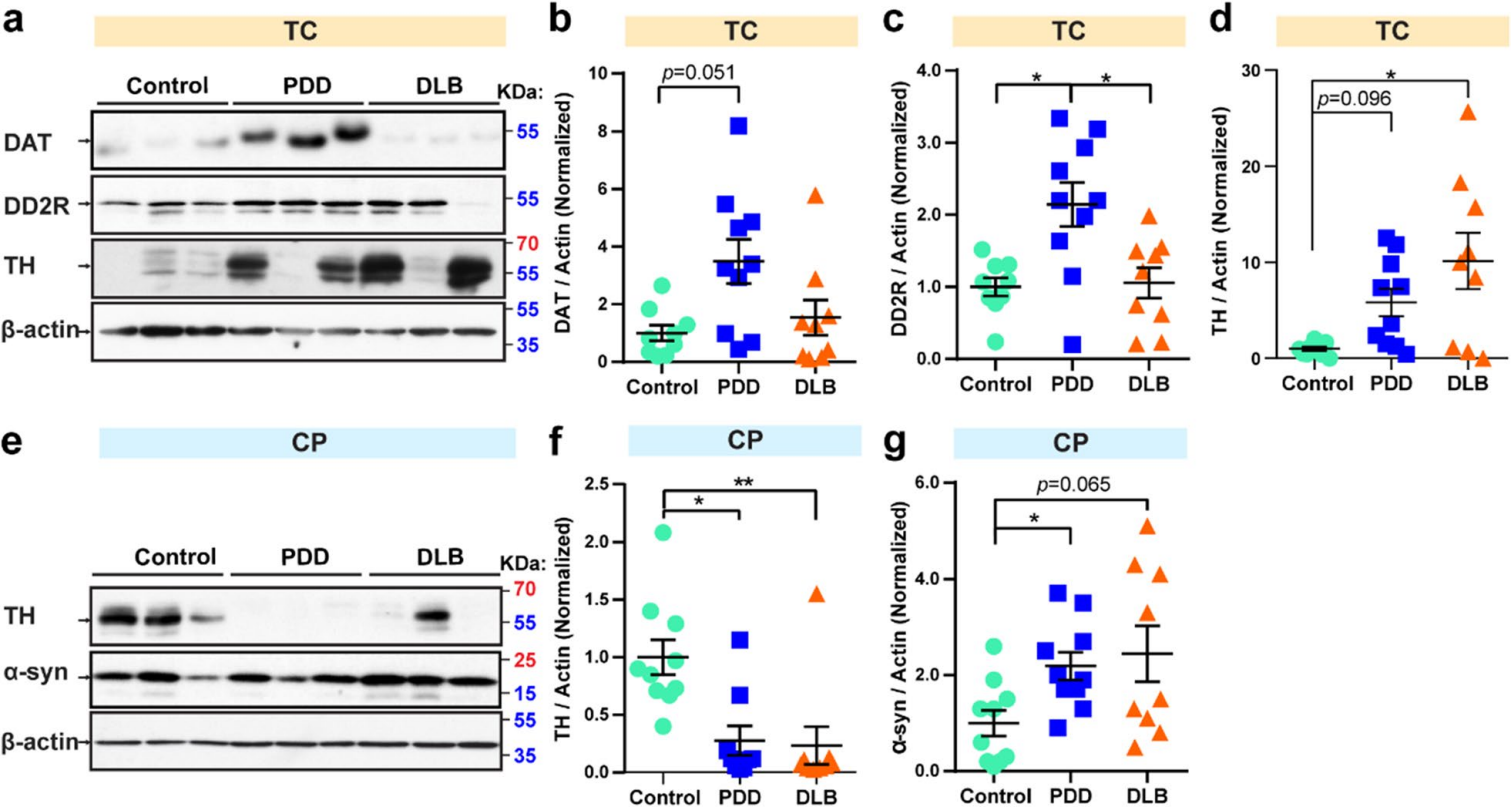
Decreased TH in both PDD and DLB but increased α-synuclein in DLB in CP. **a** Multivariate analysis of post-mortem samples from TC by western blot. Twenty micrograms of protein were used per well for SDS-PAGE. Blots were cropped from different gels. Representative western blot images were shown. Statistical results of relative protein levels for DAT (**b**), DD2R (**c**), and TH (**d**). **e** Multivariate analysis of post-mortem samples from CP by western blot. Twenty micrograms of protein were used per well for SDS-PAGE. Blots were cropped from different gels. Representative western blot images were shown. All blot images with significant changes were included in the Additional file 1: Fig. S2. Statistical results of relative protein levels for TH (**f**) and α-synuclein (α-syn) (**g**). Protein values are normalized to β-actin levels and control samples. Data are presented as the mean  ±  SEM. Kruskal–Wallis test was used to measure the differences between Control, PDD, and DLB samples. *p  < 0.05, **p  < 0.01. Sample numbers, Control  = 9, PDD  = 9–10, and DLB  = 9

PDD and DLB share many pathological features of parkinsonism. PD is characterized by a severe loss and depletion of dopamine in the substantia nigra (SN) [25]. TH participates in catalyzing the conversion of L-tyrosine to L-3,4-dihydroxyphenylalanine (L-dopamine), which is also the rate-limiting step of dopamine biosynthesis [26]. The dopamine transporter (DAT) is a transmembrane protein that moves dopamine from the synaptic cleft into the cytosol. Alpha-synuclein aggregation is the pathological hallmark of Lewy bodies and is strongly linked to DLB and PD [27, 28]. Dopamine D2 receptor (DD2R) is the D2 subtype of the dopamine receptor, and DD2R agonists are used in the treatment of PD [29]. Therefore, we studied the protein profile and distribution of these signature PD pathogenic markers by western blot analysis. In the TC region, compared to the control, DAT showed an increasing trend (by 2.5-fold, p  = 0.051) in PDD (Fig. 2a, b). DD2R protein levels were increased 1.1-fold (*p*  = 0.024) in PDD compared to control and by 97.0% (*p*  = 0.043) in PDD compared to DLB (Fig. 2a, c). Also, the TH levels were significantly elevated in DLB (by 9.1-fold, *p*  = 0.038) and showed an increasing trend in PDD (by 4.84-fold, *p*  = 0.096) compared to control (Fig. 2a, d). Notably, in the CP region, TH levels were significantly decreased in both PDD (by 72.3%, *p*  = 0.021) and DLB (by 76.6%, *p*  = 0.003) compared to control (Fig. 2e, f). Besides, α-synuclein levels were increased by 1.2-fold (*p*  = 0.049) in PDD and showed an increasing trend in DLB (by 1.7-fold, *p*  = 0.065) as compared to control (Fig. 2e, g). No significant difference of PD-related protein was detected in the SN region (Table 3 and discussed in the “Discussion” section). These data demonstrate that level of PD pathogenic protein TH is decreased and α-synuclein is increased in both PDD and DLB in the CP region, reflective of the parkinsonism feature in both diseases. The different expression levels of TH in the TC and CP region may indicate its region-specific expression pattern in the brain of the two disease profiles, PDD and DLB.

**Table 3:** Statistic results of PD-related protein markers tested in three regions of Control (Ctrl), PDD, and DLB post-mortem samples

Protein	Region	PDD/Ctrl	DLB/Ctrl	PDD/DLB
TH	SN	NS	NS	NS
	CP	↓* (0.021)	↓** (0.003)	NS
	TC	↑ (0.096)	↑* (0.038)	NS
DAT	SN	NS	NS	NS
	CP	NS	NS	NS
	TC	↑ (0.051)	NS	NS
α-Syn	SN	NS	NS	NS
	CP	↑* (0.049)	↑ (0.065)	NS
	TC	NS	NS	NS
p-α-Syn^S129^	SN	NS	NS	NS
	CP	NS	NS	NS
	TC	NS	NS	NS
DD2R	SN	NS	NS	NS
	CP	NS	NS	NS
	TC	↑* (0.024)	NS	↑* (0.043)

Statistical results from western blot results for PD-related protein markers tested in this study. Western blot images were quantified using ImageJ, and the data were normalized to β-actin. The upwards arrow indicates an increase and the downwards arrow indicates a decrease. *p*  < 0.1 was included in the bracket to show the trends of the protein levels. Kruskal–Wallis test was used to measure the differences between Control, PDD, and DLB samples

*NS *no significance

*p  < 0.05 and **p  < 0.01

### Neuroinflammation and mitophagy activity is increased in PDD

**Fig. 3:**
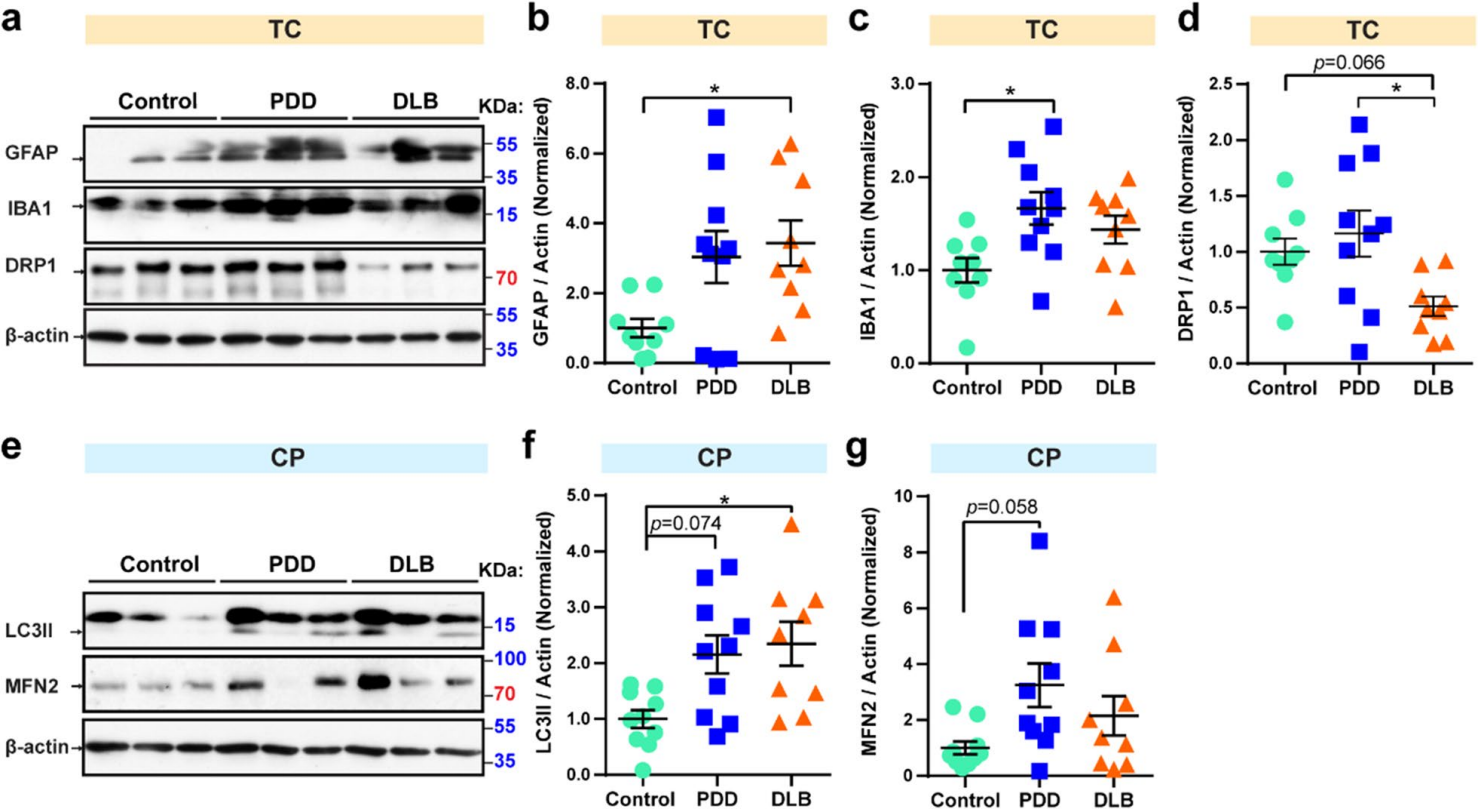
Increased neuroinflammation, mitophagy activity, and mitochondrial dynamics in PDD. **a** Multivariate analysis of post-mortem samples from TC by western blot. Twenty micrograms of protein were used per well for SDS-PAGE. Blots were cropped from different gels. Representative western blot images were shown. Statistical results of the relative protein levels of GFAP (**b**), IBA1 (**c**), and DRP1 (**d**). **e** Multivariate analysis of post-mortem samples from CP by western blot. Blots were cropped from different gels. Representative western blot images were shown. All blot images with significant changes were included in the Additional file 1: Fig. S3. Statistical results of the relative protein levels of LC3II (**f**), and MFN2 (**g**). Protein values are normalized to β-actin levels and control samples. Data are presented as the mean  ±  SEM. Kruskal–Wallis test was used to measure the differences between Control, PDD, and DLB samples. *p  < 0.05. Sample numbers, Control  = 9, PDD  =  10, and DLB  = 9

Chronic neuroinflammation is one of the pathophysiological hallmarks of PD and α-synuclein pathology [30]. Microglia are involved in neuroinflammation in the central nervous system [31]. Mitochondria provide an energy source for cells; thus, mitochondrial dynamics are vital for cell survival and function. Mitophagy is the selective removal of impaired mitochondria via autophagosomes and digestion by lysosomes [32]. The removal of damaged mitochondria is very important, and mitophagy is linked to neurodegenerative disease [33]. To investigate the correlation of neuroinflammation, mitophagy, and mitochondrial dynamics with PDD and DLB, their representative protein markers were studied. In the TC region, compared to control samples, the level of the astroglia marker GFAP, which is related to the astrocytic response, was increased in DLB samples (by 2.4-fold, *p*  = 0.025) (Fig. 3a, b). The level of neuroinflammation marker IBA1, related to microglial activation, was enhanced in PDD (by 66.5%, *p*  = 0.020) compared to control samples (Fig. 3a, c). As a mitochondrial dynamic network component, DRP1 level was increased by 1.3-fold (*p* = 0.016) in PDD samples compared to DLB samples (Fig. 3a, d). In the CP region, compared to control samples, as a mitophagy marker, LC3II protein levels were increased in DLB samples (by 1.3-fold, *p * = 0.040) and showed an increasing trend in PDD samples (by 1.2-fold, *p * = 0.074) (Fig. 3e, f). Besides, compared to control, MFN2 has an increasing trend in PDD samples (by 2.3-fold, *p * = 0.058) (Fig. 3e, g). Collectively, our results suggest that neuroinflammation and mitophagy are generally enhanced in PDD and play important role in neurodegeneration (Table 4).

**Table 4:** Statistic results of mitochondrial, neuroinflammation, and autophagy-related protein markers tested in three regions of Control (Ctrl), PDD, and DLB post-mortem samples

Protein	Region	PDD/Ctrl	DLB/Ctrl	PDD/DLB
GFAP	SN	NS	NS	NS
	CP	NS	NS	↑ (0.075)
	TC	↑ (0.060)	↑* (0.025)	NS
Iba1	SN	NS	NS	NS
	CP	NS	NS	NS
	TC	↑* (0.020)	NS	NS
LC3II	SN	NS	NS	NS
	CP	↑ (0.074)	↑* (0.040)	NS
	TC	NS	NS	NS
p62	SN	NS	NS	NS
	CP	NS	NS	NS
	TC	NS	NS	↑ (0.052)
Tom20	SN	NS	NS	NS
	CP	NS	NS	NS
	TC	NS	NS	NS
DRP1	SN	NS	NS	NS
	CP	NS	NS	NS
	TC	NS	↓ (0.066)	↑* (0.016)
MFN2	SN	NS	NS	NS
	CP	↑ (0.058)	NS	NS
	TC	NS	NS	NS

Statistical results from western blot results for mitochondrial, neuroinflammation and autophagy-related protein markers were investigated in this study. Western blot images were quantified using ImageJ, and the data were normalized to β-actin. The upwards arrow indicates an increase and the downwards arrow indicates a decrease. *p*  < 0.1 was included in the bracket to show the trends of the protein levels. Kruskal–Wallis test was used to measure the differences between Control, PDD, and DLB samples

*NS* no significance

*p  < 0.05

### Expression of p-LRRK2^S935^ is increased in PDD in the TC region

**Fig. 4:**
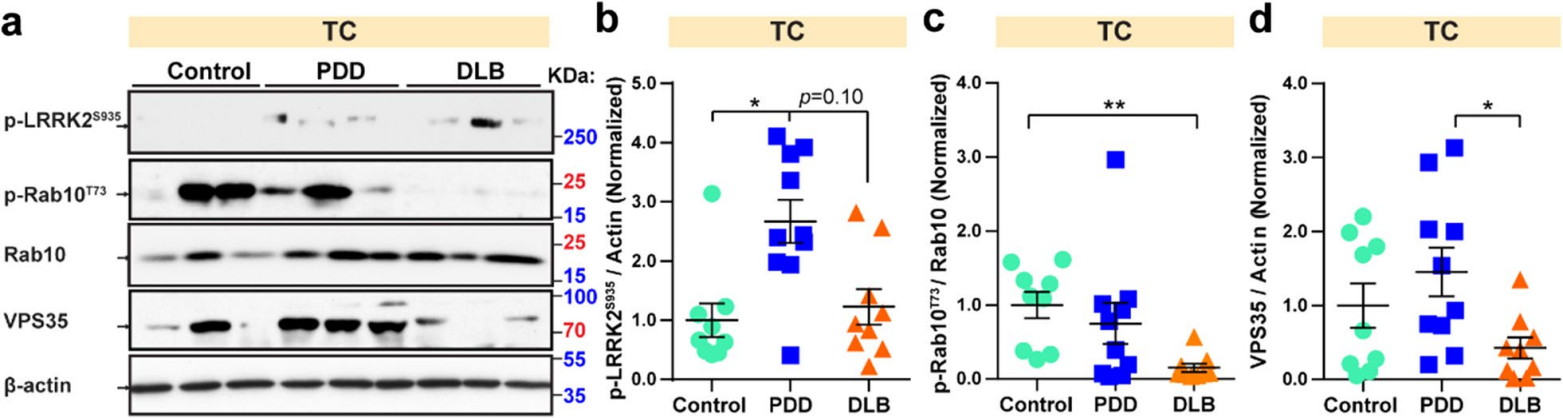
Increased p-LRRK2^S935^ in PDD and increased VPS35 in PDD compared to DLB in TC. **a** Multivariate analysis of post-mortem samples from the TC by western blot. Twenty micrograms of protein were used per well for SDS-PAGE. Blots were cropped from different gels. Representative western blot images were shown. All blot images with significant changes were included in the Additional file 1: Fig. S4. Statistical results of the relative protein levels of p-LRRK2^S935^ (**b**), p-Rab10^T73^/Rab10 (**c**), and VPS35 (**d**). Data are presented as the mean  ±  SEM. Kruskal–Wallis test was used to measure the differences between Control, PDD, and DLB samples. *p  < 0.05, **p  < 0.01. Sample numbers, Control  = 9, PDD  = 10, and DLB  = 9

Mutations in leucine-rich repeat kinase 2 (LRRK2) are a common genetic cause of both familial and sporadic PD [34]. We have reported that mutant LRRK2 can phosphorylate APP at Thr-668 in the APP intracellular domain (AICD), which increases the nuclear transcription of AICD, leading to dopaminergic neuron loss [35, 36]. This mutation also links the pathologies of PD and AD, which share the common feature of dementia. To further investigate the function of LRRK2 and its substrates on PDD and DLB, we examined the protein expression of LRRK2 and its substrates, including VPS35, Rab10, and p-Rab10^T73^ [37–39]. Rab10 is a LRRK2 substrate and Rab10^T73^ phosphorylation is proposed to be a valid target in LRRK2-related PD [40]. In the TC region, compared to control samples, the level of p-LRRK2^S935^ was enhanced (by 1.7-fold, *p*  = 0.023) in PDD but not in DLB (Fig. 4a, b). The relative level of p-Rab10^T73^ to Rab10 in DLB was decreased by 84.9% (*p*  = 0.002) in DLB compared to control (Fig. 4a, c). VPS35 levels in PDD were found to be 2.4-fold higher (*p*  = 0.037) than DLB (Fig. 4a, d). Notably, LRRK2 substrates did not show statistical changes in SN and CP regions (Table 5). Taken together, our results suggest that level of p-LRRK2^S935^ is increased in PDD compared to control and LRRK2 substrates seem to be higher in PDD than in DLB in the TC region.

**Table 5:** Statistic results of LRRK2-related proteins tested in three regions of Control (Ctrl), PDD, and DLB post-mortem samples

Protein	Region	PDD/Ctrl	DLB/Ctrl	PDD/DLB
LRRK2	SN	NS	NS	NS
	CP	NS	NS	NS
	TC	NS	NS	NS
p-LRRK2^S935^	TC	↑* (0.023)	NS	NS
Rab10	SN	NS	NS	NS
	CP	NS	NS	NS
	TC	↑*** (0.0006)	↑** (0.002)	NS
p-Rab10^T73^/Rab10	TC	NS	↓** (0.002)	NS
VPS35	SN	↑ (0.067)	NS	NS
	CP	NS	NS	NS
	TC	NS	NS	↑* (0.037)
14-3-3	SN	NS	NS	NS
	CP	NS	NS	NS
	TC	NS	NS	NS

Statistical results from western blot results for LRRK2-substrate proteins tested in this study. Western blot images were quantified using ImageJ, and the data were normalized to β-actin. The upwards arrow indicates an increase and the downwards arrow indicates a decrease. *p*  < 0.1 was included in the bracket to show the trends of the protein levels. Kruskal–Wallis test was used to measure the differences between Control, PDD, and DLB samples

*NS *no significance

*p  < 0.05, **p  < 0.01, ***p  < 0.001

## Discussion

In this study, to understand the difference in molecular mechanisms between PDD and DLB, we systematically studied a variety of protein markers, including those found in AD and PD pathogenesis and neuroinflammation, microglia, mitophagy, and mitochondria-related markers, using human post-mortem brain tissues from individuals with PDD and DLB. Although the clinical and pathological features of each case are different even in the same disease condition group (Additional file 1: Table S1), we are still able to detect some common protein changes. Our results showed similar pathological features in both diseases, such as increased p-APP^T668^ in TC, as well as decreased TH, and increased α-syn and LC3II in the CP region. The similar expression patterns of these pathological proteins reflect the common clinical presentations shared by both diseases, which may help to understand the mechanism underlying the common pathological features of PDD and DLB. Notably, we also detected differences between the PDD and DLB. We found that DD2R, DRP1, and VPS35 in the TC region and p-Tau^S199/202^ in the CP region are higher in PDD than DLB. Most proteins tests showed increased expression in PDD samples, which implies PDD is a progressive forward disease. While in DLB samples, those protein markers showed a complex pattern, which may contribute to the difficulty in the diagnosis of DLB. Taken together, this set of signature proteins could be useful to study the different etiology and pathogenesis of PDD and DLB.

Amyloid deposits and Tau tangles are the signature features of dementia related to AD. We found that Aβ42 levels in the TC region were increased in PDD samples, but no difference in these levels was observed in DLB samples compared to control (Fig. 1a). Studies have reported that Aβ42 levels are lower in samples from individuals with PDD or DLB [41–44]. All of these studies measured Aβ42 levels in CSF samples, which may not directly reflect the actual Aβ42 levels in the brain. Higher Aβ42 levels may exist in the cortex of patients with PDD, thus leading to the accumulation of amyloid protein in the cortex, with fewer Aβ42 being released into the CSF. Recent studies showed that Aβ plaque load is overall higher in the DLB brain and the distribution of Aβ varies in different sub-brain regions [45]. A proportion of DLB patients have low Aβ plaque load, compared to the control group, but the Aβ-negative DLB group still exhibited cortical thinning in certain brain regions, such as entorhinal, basal frontal, and occipito-parietal cortices [46]. Our study did not detect a difference in Aβ load between the DLB and control group, which may be due to the presence of some low Aβ load DLB patients. From Additional file 1: Table S1, we found that certain control samples also have a mild load of Aβ related pathology in the frontal, temporal and entorhinal cortices, while some DLB cases have diffuse and neuritic plaques in the superior frontal gyrus but with no significant tau-related pathology.

Our study also showed an increased trend of p-APP^T668^ levels in the TC region of both individuals with PDD and those with DLB (Fig. 1b, c). Increased p-APP^T668^ levels in the brain reflect the pathological feature of PDD, which is a common signature feature of dementia pathology. Interestingly, p-Tau^S199/202^ levels were not changed in PDD samples in the CP region and also in TC and SN regions (Fig. 1d, e and Table 2); however, they were significantly decreased in the CP region in DLB samples compared to both control and PDD samples (Fig. 1d, e). This indicates that increased expression of AD pathogenesis marker p-Tau^S199/202^ is higher in PDD compared to DLB. Interestingly, a study on p-Tau^S199/202^ levels in the CSF of individuals with PDD and DLB revealed that p-Tau^S199/202^ level is higher in the CSF of individuals with DLB [47]. We investigated the phosphorylation of Tau at Ser-199/202 sites using post-mortem brain samples from the CP region, while Anderson and colleagues used phosphorylated Tau at Thr-181 using CSF samples [47]. Different phosphorylation patterns may exist in Tau at Thr-181 and Ser-199/202 sites or between CSF and CP brain tissue samples. Also, Anderson et al*.*’s detection of p-Tau^T181^ protein levels was performed using ELISA, which may include nonspecific binding to phospho-Tau at Thr-181. In this study, our findings showed increased p-Tau^S199/202^ level in the CP region, which could be used to distinguish PDD from DLB. Figure 1 showed p-Tau ^S199/202^ level was relatively lower in DLB than in control and PDD groups, which may due to the regional heterogeneity of p-Tau level in the human brain. A recent study also reported differentially expressed protein levels in diverse brain areas of PD and AD patients. They showed that the relative p-Tau levels are lower in SN and cortex areas but higher in the hippocampus region of PD samples [48].

PDD and DLB share similar clinical and parkinsonism features, such as limb rigidity, tremor, cognitive impairment, and Lewy body formation [1]. To understand the expression pattern of PD pathogenic markers in both PDD and DLB brains, we thus investigated classic PD-related proteins, including TH, DAT, α-synuclein, and the DD2R in human post-mortem brain tissue samples. Our results showed that TH was lower and α-synuclein was higher in both PDD and DLB in the CP region (Fig. 2e–g), which reflects the parkinsonian feature seen in PDD [26, 28]. Interestingly, the TH level seems higher in PDD and DLB in the TC region (Fig. 2a, d). The different TH levels in TC and CP zone suggest region-specific expression of TH in the brain, which was also reported in a study in rats exposed to lead [49]. Although DAT is important for maintaining dopamine levels in the cell, an imaging study looking at DAT levels in post-mortem PD brain samples did not show a correlation between nigral neurons and PD [50]. This is similar to our findings showing no significant difference in DAT between SN samples in PDD brain samples and those of controls. PD patients are usually diagnosed with decreased DAT and DD2R levels in the SN zone. One group of researchers detected overactivation of the prefrontal lobe in PD patients during obstacle negotiation [51]. They explained that the saturated prefrontal cortex may hinder patients from performing other tasks. Similarly, one possible explanation is that DAT and DD2R have different distribution in different brain regions in PDD; thus, oversaturated DAT and DD2R in the TC region may block the normal transmission of dopamine neurons.

We see the variability of some protein levels such as TH, p-APP, and p-Rab10^T73^ from individual post-mortem brains, which is commonly observed in similar studies [14, 48]. The main reason may be due to the heterogeneity of dopamine neurons in different brain regions (reviewed in [52–54]). Dopamine neurons are not homogenous in distinct anatomical brain regions and different populations have different pathophysiological properties [54–56]. First, dopaminergic neurons have a large axonal projection area, which may span different brain regions [57, 58]. Second, it is a common observation in multiple neurodegeneration diseases that TH may lose immunoreactivity without a concomitant loss of dopamine neurons [59, 60]. Next, the dopamine transporter (DAT), which reuptakes presynaptic dopamine neurons at the synapse, is detectable only in about half of the ventral tegmental neurons [61]. Lastly, the dopamine D2 receptor (DD2R) acts in a negative feedback loop to reduce neuronal firing when activated by extracellular dopamine [62]. The DD2R activation threshold and effect on extracellular dopamine levels vary significantly between brain regions [63, 64]. Therefore, because of the complexity of dopamine cycling regulated by TH, DAT, and DD2R as well as large axonal projection areas in different brain regions, dopamine neuron, and dopamine-related protein levels vary in different regions and individuals.

Neuroinflammation, mitophagy activity, and mitochondrial dynamics are thought to be associated with PD and DLB. Our results showed that levels of both GFAP and IBA1, markers of neuroinflammation, were generally increased in the TC region in DLB and PDD (Fig. 3), suggesting that neuroinflammation is upregulated in both PDD and DLB pathology, which is consistent with a previous report [30]. Mitophagy activity is also found to be enhanced in both PDD and DLB in the CP region, marked by higher levels of LC3II (Fig. 3e, f). This result could represent the feedback mechanism of mitophagy, in which mitophagy activity is stimulated by elevated levels of impaired mitochondria in cells.

LRRK2 regulates vesicle trafficking by phosphorylating the Rab family of proteins, while the cargo-binding component of the retromer complex VPS35 mutation enhances this activity in *Drosophila*, mouse, and human models [37, 38]. Our results show that both p-LRRK2^S935^ and VPS35 protein levels were or tended to increase in PDD samples compared to DLB (Fig. 4), which is consistent with previous findings that PD-linked VPS35 mutations can induce dopaminergic neurodegeneration [65]. VPS35 levels are not changed in DLB compared to control, suggesting that VPS35 levels are independent of Lewy body pathology. Surprisingly, we also found that the relative p-Rab10^T73^ level is decreased in DLB samples when compared to the control group (Fig. 4). The different expression levels of p-LRRK2^S935^ and LRRK2 substrates suggest the contrasting roles of LRRK2 in PDD and DLB pathology, thus signifying their potential in differentiating PDD and DLB.

One of our interesting findings is that all the pathogenic proteins of AD and PD, as well as neuroinflammation and mitophagy, investigated in this study only showed significant changes in the TC and CP regions but not in the SN region (Tables 2, 3, 4, 5). This result may reflect the progression and stage of the disease. Dementia is the most prominent common feature between PDD and DLB. In the early stage of PD, movement disorder symptoms are more severe than dementia symptoms, and pathogenic protein changes may be more prone to occur in the SN [66]. However, in the later stage, dementia becomes more severe, and there is more neuronal loss in the cerebral cortex. Therefore, more signature proteins were dysregulated in the TC and CP regions of the brain at this time point. Another possible reason is that although substantia nigra is believed to be the most active area for dopamine neurons, high heterogeneity still exists due to the large axonal projection area and the dynamic cycle of dopamine neurogenesis. This may lead to the variation in dopamine-related protein levels such that no significant difference could be detected in the SN zone.

A systematic study of different subsets of protein levels was measured in TC, SN, and CP zones of PDD and DLB samples, and compared to control. Although several showed that significant changes were detected, the majority of proteins did not show significant changes in all three regions of brain tissue (Tables 2, 3, 4, 5). Heterogeneity of protein expression patterns was also observed in some proteins. One possible reason is the broad age range and symptom duration of the subjects in this study. The age of the control group ranges across about 30 years for both controls and DLB groups (Table 1). The symptom duration ranges from 5 to 34 years within the PDD group and 1–15 years within the DLB group (Table 1). Reports have revealed mRNA and protein levels change with the aging process, especially for some processes such as oxidative stress and mitochondrial functions [67–69]. Therefore, many protein levels detected were diverse even within the same group, and hardly reached statistical significance among different groups. Another possible reason is that, although the main cause of death of the control subjects was not neurological-related, some of the control subjects indeed had some mild neurodegenerative-related presentations, such as tau pathology and low AD neuropathologic change (Additional file 1: Table S1). Some pathologic signature proteins may be largely different from other subjects within the control group. Nevertheless, even with diverse protein expression patterns of some signature proteins, a series of differentially expressed proteins were captured among controls, PDD, and DLB, which illustrates the pathologic features of PDD and DLB.

## Conclusions

Our comprehensive and systematic protein expression study has provided additional information relating to PDD and DLB pathology, yet it has its limitations. First, the sample size is small due to the limited availability of patient post-mortem samples. However, more than twenty proteins were measured in all three regions of the brain in every sample. The systematic study of the set of proteins simultaneously in the same brain disease condition will provide a better understanding of the spatial distribution of proteins underlying the pathogenesis and etiology of PDD and DLB at the molecular level. Next, the signature proteins detected in the brain may not reflect their CSF or blood levels or even be present in the biofluid circular system, thus it could not directly be used as biomarkers for the diagnosis of diseases in clinical practice. Future CSF or blood studies will warrant if they can be used as biomarkers in clinical practice. In summary, our comprehensive and systematic study identified a set of signature neurobiological markers in PDD and DLB, which will help to understand the pathologic features of DLB and PDD at the molecular level, which is vital for accurate clinical diagnosis and future therapeutic development.

## Materials and methods

### Brain tissues

Frozen brain tissues, including SN, TC, and CP regions, that were used in this study were obtained from the Parkinson’s UK Brain Bank, Division of Neuroscience, Imperial College London. Nine brain samples from controls (six males and three females), 10 samples from individuals with PDD (six males and four females), and nine samples from individuals with DLB (six males and three females), whose ages ranged from 58 to 98, were used in this study. Subjects with non-neurological or low clinical AD presentation were considered as controls for PDD and DLB and their post-mortem brain tissues were used in this study. All tissues were obtained via a prospective donor scheme with fully informed written consent and their collection was approved by the UK Human Tissue Authority (Approval Number 18/WA/0238). All methods and protocols used in this study were performed in accordance with the institutional and relevant guidelines and regulations. The cause of death, clinical diagnosis, and pathological feature of each case were carefully examined by the clinical doctors and pathologists. This study was also approved by the Institutional Review Board (IRB) of the National Neuroscience Institute of Singapore.

### Protein extraction from brain tissue

For western blot, proteins were extracted from approximately 50 mg of snap-frozen brain tissues using lysis buffer I (10 mM Tris–Cl pH 8.0, 1 mM EDTA, 0.1% Triton X-100, 0.1% SDS, 250 mM NaCl). For ELISA assay, snap-frozen brain tissues were lysed in lysis buffer II (10 mM Tris–Cl pH 8.0, 1 mM EDTA, 1% Triton X-100, 250 mM NaCl). The lysis buffers were supplemented with a proteinase inhibitor cocktail (MCE, HY-K0010) and a phosphatases inhibitor cocktail (MCE, HY-K0022). Briefly, the snap-frozen brain samples were ground using a plastic pestle driven by a micromotor. After incubation on ice for 30 min and centrifugation at 12,000*g* for 15 min at 4 °C, the supernatant was collected for the experiments. The protein concentration was determined by the DC™ Protein Assay Kit (Bio-Rad, 5000122).

### ELISA assay

One hundred micrograms of each TC protein sample were used for the enzyme-linked immunosorbent assay (ELISA) to detect Aβ42 peptide. The ELISA assay was performed according to the manufacturer’s manual of the ELISA kit (FUJIFILM, 296-64401). Briefly, a 100 mg protein sample in 100 μl was dispensed into the antibody-coated microplate provided in the kit and left overnight at 4 °C after sealing the plate. The microplate was incubated with an HRP-conjugated antibody in the refrigerator for overnight after washing. The stop solution was added to the wells after incubation for 20 min with the TMB (3,3′,5,5′-Tetramethylbenzidine) solution. The Aβ42 concentrations were measured by a microplate reader at 450 nm.

### SDS-PAGE and immunoblotting

Twenty micrograms of each protein sample were loaded onto a sodium dodecyl sulfate–polyacrylamide gel electrophoresis (SDS-PAGE) gel for separation using a Bio-Rad Mini-PROTEAN^®^ System. To separate and show both low and high molecular weight proteins, three layers of gels (from top to bottom, 4–8–15%) were used to cast each SDS-PAGE gel. Due to the size limit, we could not load and compare all 28 samples on a single gel. We separated all samples into three groups, and each group contained three control samples, four PDD samples, and three DLB samples, balanced for age and gender, that were loaded onto three SDS-PAGE gels at the same time. For each set of SDS-PAGE and western blot runs, three gels containing all 28 samples were run in parallel. Beta-actin was used as the internal control for the actual protein amount. Proteins were transferred onto a 0.45 μm polyvinylidene difluoride (PVDF) membrane (Millipore, #IPVH00010) for immunoblotting. Since we had many antibodies to detect in every sample and the total protein amount for each sample is limited, we had to cut each PVDF membrane into 3 or 4 pieces to separate 3 to 4 protein fractions for separate immunoblotting with different antibodies according to the protein standard (Thermo Fisher, #26619). The PVDF membrane was blocked with 5% skimmed milk in TBS (Tris-buffered saline) buffer containing 0.1% Tween-20 (Sigma, P1379) for 1 h and was incubated overnight with primary antibodies diluted with 1 × PBS TBST buffer after three washes. The antibodies used in this study were p-Tau^S199/202^ (Millipore, #ab9674), APP (Calbiochem, #171610), p-APP^T668^ (Cell Signaling, #3823), LRRK2 (Sigma, #L9918), p-LRRK2^S935^ (Abcam, #ab133450), tyrosine hydroxylase (Millipore, #MAB318), α-synuclein (BD Transduction Lab, #610787), dopamine transporter (Millipore, #2231), dopamine D2 receptor (Millipore, #AB5084P), VPS35 (Abcam, #ab157220), β-actin (Santa Cruz, #AC-15), p-α-synuclein (Ser129) (Cell Signaling, #23706), LC3II (Abcam, #ab243506), p62 (Cell Signaling, #39749), Tom20 (Cell Signaling, #42406), DRP1 (Cell Signaling, #8570), mitofusin-2 (Cell Signaling, #9482), Rab10 (Cell Signaling, #8127), p-Rab10^T73^ (Abcam, #230261), 14-3-3 (Cell Signaling, #9636), CD11b (Bio-Rad, #MCA275R), GFAP (Abcam, #AB68428), anti-mouse IgG HRP (GE Healthcare, #NA931V), and anti-rabbit IgG HRP (GE Healthcare, #NA934V). The blotting membranes were incubated with secondary antibodies for 2 h before developing. Membranes were developed by Pierce™ ECL western blotting substrate (Thermo Fisher, #32106) or SuperSignal™ West Femto Maximum Sensitivity Substrate (Thermo Fisher, #34095) in an X-ray machine.

### Image processing and statistical analyses

The intensity of each sample was measured and quantified using ImageJ software. The relative protein amount for each sample was normalized to its β-actin protein amount. Then, the relative protein amount was further normalized to the average protein amount for the control group. All statistical analyses and figures were performed and created using GraphPad Prism software. A two-tailed student’s *t *test with equal variance was used to analyze statistical differences between the two groups. Since protein expression levels in each case might not be normally distributed, the non-parametric Kruskal–Wallis test was used to compare the differences between each disease group for all other data sets. The statistical significance levels were set at *p  < 0.05, **p  < 0.01, and ***p  < 0.001. For the control sample, n  = 9; for the PDD sample, n  = 10; and for the DLB sample, n  = 9.

## Supplementary Information

**Additional file 1.** Supplementary table and figures.

## Data Availability

The datasets used in the current study are available from the corresponding author on reasonable request.

## Abbreviations

AD: Alzheimer’s disease
AICD: Amyloid precursor protein intracellular domain
CP: Caudate and putamen
DAT: Dopamine transporter
DD2R: Dopamine D2 receptor
DLB: Dementia with Lewy bodies
L-Dopamine: L-3,4-dihydroxyphenylalanine
LRRK2: Leucine-rich repeat kinase 2
MFN2: Mitofusin-2
PD: Parkinson’s disease
PDD: Parkinson’s disease dementia
PINK1: PTEN induced putative kinase 1
SN: Substantia nigra
TC: Temporal cortex
TH: Tyrosine hydroxylase
