# Supplementary material for: Increased expression of pathological markers in Parkinson’s disease dementia post-mortem brains compared to dementia with Lewy bodies
Source: BMC Neurosci. 2022 Jan 4;23:3. doi: 10.1186/s12868-021-00687-4 (PMC8725407; doi:10.1186/s12868-021-00687-4)
Supplement: Supplementary file 1 — Additional file 1. Supplementary table and figures. [file 12868_2021_687_MOESM1_ESM.docx]

**Increased Expression of Pathological Markers in Parkinson’s Disease Dementia Post-Mortem Brains Compared to Dementia with Lewy Bodies**

Haitao Tu^1^, Zhiwei Zhang^1^, Lifeng Qiu^1^, Yuning Lin^2^, Mei Jiang^1,3^, Sook-Yoong Chia^1^, Yanfei Wei^2^, Adeline SL Ng^4,5^, Richard Reynolds^6,7^, Eng-King Tan^4,5^, Li Zeng^1,5,7*^

^1^Neural Stem Cell Research Lab, Research Department, National Neuroscience Institute, Singapore 308433.

^2^Guangxi University of Chinese Medicine, 179 Mingxiu Dong Rd. Nanning, Guangxi, China 530001.

^3^Department of Anatomy and Neurobiology, Zhongshan School of Medicine, Sun Yat-Sen University, #74, Zhongshan No. 2 Road, Guangzhou, China 510080.

^4^Department of Neurology, National Neuroscience Institute, Singapore 308433.

^5^Neuroscience & Behavioral Disorders Program, DUKE-NUS Graduate Medical School, Singapore 169857.

^6^Division of Neuroscience, Imperial College London, Hammersmith Hospital, London W12 0NN.

^7^Centre for Molecular Neuropathology, Lee Kong Chian School of Medicine, Nanyang Technological University, Novena Campus, 11 Mandalay Road, Singapore 308232.

*** Corresponding Authors:**

Tel: (65) 6357 7515

Li ZENG, [Li_Zeng@nni.com.sg](mailto:Li_Zeng@nni.com.sg)

**Supplementary Table 1. Clinical diagnosis and Pathological features of cases used in this study.**

| **Cases** | **Sex** | **Age** | **Onset** | **Cause of death** | **Clinical diagnosis** | **Braak stage** | **Alpha-synuclein pathology** | **Tau and Aβ pathology** |
| --- | --- | --- | --- | --- | --- | --- | --- | --- |
| **Ctrl #1** | M | 66 | N.A. | Pancreatic cancer | - Mild age-related changes and mild hypoxia-related features | N.A. | - No α-synuclein pathology | - No tau-related pathology. - Mild load of Aβ related pathology in the frontal, temporal and entorhinal cortices - No plaques in the primary visual cortex. |
| **Ctrl #2** | F | 70 | 59 | Respiratory failure & atypical Parkinson’s disease | - Alzheimer-type tau pathology | Braak stage I | - No α-synuclein pathology | - Iso and allocortical Aβ pathology and tau pathology mainly restricted to the entorhinal cortex. - Up to moderate CAA type 2 in the occipital lobe. |
| **Ctrl #3** | M | 76 | N.A. | Chronic obstructive pulmonary disease | - Age associated changes | Braak stage I | - No α-synuclein pathology | - Iso and allocortical parenchymal Aβ pathology and mild tau pathology restricted to the entorhinal cortex - CERAD negative - Low AD neuropathologic change |
| **Ctrl #4** | M | 87 | N.A. | Conductive Cardiac Failure. Chronic kidney disease. | - tau pathology consistent with argyrophilic grain disease | N.A. | - No α-synuclein pathology | - Many Aβ plaques in the anterior frontal and insular cortices - Fewer plaques in the occipital, entorhinal and fusiform cortices - A few tau tangles and neuritic staining in the basal forebrain - Occasional glial deposits - Occasional tangles in the CP - Occasional tangles in the rostral SN |
| **Ctrl #5** | M | 90 | N.A. | Respiratory failure secondary to bronchial cancer | - Mild age-related and microvascular changes | N.A. | - No α-synuclein pathology | - A few small diffuse deposits in the occipital lobe, no plaques seen - A few p-tau tangles in the amygdala and more in temporal cortex - Neurofibrillary tangles are seen in the hippocampus - Tangles in the transentorhinal and entorhinal cortex. - Tangles are negative in midbrain, pons and medulla |
| **Ctrl #6** | M | 91 | N.A. | Unknown | - Low AD neuropathologic change - PART - ARTAG | Braak stage II | - No α-synuclein pathology | - Isocortical amyloid pathology, mainly allocortical tau pathology - CERAD negative - Low AD neuropathologic change - Possible PART - ARTAG |
| **Ctrl #7** | F | 92 | N.A. | Unknown | - Low AD neuropathologic change (age associated changes) | Braak stage III | - No α-synuclein pathology | - Iso and allocortical amyloid and mainly allocortical tau pathology - Moderate CAA type 1 - CERAD negative - NIA-AA score: A2, B2, C0 - Low AD neuropathologic change; |
| **Ctrl #8** | F | 98 | N.A. | Old age | - Low AD neuropathologic change | Braak stage III | - No α-synuclein pathology | - Iso and allocortical amyloid pathology and mainly allocortical tau pathology - Mild CAA type 2 - CERAD, A - NIA-AA: A2, B2, C1 - Low AD neuropathologic change |
| **Ctrl #9** | M | 79 | N.A. | Brainstem stroke, bronchopneumonia | - Low AD neuropathologic change. - Possible PART | Braak stage II | - No α-synuclein pathology | - Sparse isocortical amyloid pathology and mainly allocortical tau pathology - NIA-AA: A1, B1, C0 - Low AD neuropathologic change - Possible PART |
| **PDD #1** | F | 85 | 67 | Bronchopneumonia, Breast Cancer with metastasis and PD | - LBD, brain-stem predominant - Clinical PD - Prominent hallucinations and probable cognitive impairment. | Entorhinal grade 3 and isocortical grade 1 Aβ /tau pathology | - LBs in several nerve cells. - Unremarkable in the motor cortex, parietal lobule, temporal lobe, visual cortex and thalamus - Alpha-synuclein positive cells in hypothalamus | - Many Aβ plaques in the superior frontal gyrus but no tau tangles. - Aβ plaques in the subiculum and entorhinal cortex. - Tau-positive neurofibrillary tangles in the hippocampal, subicular and entorhinal cortex - Numerous tau-positive tangles in temporal cortex near the amygdala |
| **PDD #2** | M | 75 | 57 | Unknown | - LBD, brain-stem predominant - Clinical PD - Dementia with hallucinations | Entorhinal grade 2 and isocortical grade 1 Aβ /tau pathology | - A few LBs in the grey matter, hippocampal formation and the entorhinal cortex - A few nerve cells in the nucleus accumbens, hypothalamus and amygdala also contain α-synuclein positive deposits - No significant pathological changes in CP - Some LBs in SN | - Numerous Aβ plaques in the superior frontal gyrus but no tau - Moderate plaques in the subiculum and entorhinal cortex - Only a few tau-positive tangles in the nerve cells of the entorhinal cortex. |
| **PDD #3** | M | 75 | 42 | Unknown | - Young onset PD - LBD, brain-stem predominant - Findings consistent with long-standing clinical PDD | Entorhinal grade 3 and isocortical grade 4 Aβ /tau pathology | - Some LBs in superior frontal, mamillo-hypothalamic area and thalamus - A few positive macrophages in CP - Large LBs in the nucleus basalis of Meynert - Some activated microglial cells, macrophages, and LBs in primary visual cortex and midbrain - Loss of nerve cells in SN - Some LBs in SN and midbrain. | - Classical neuritic plaques in the superior frontal gyrus but no tau - CA2/3 has tau positive structures - Moderate number of tangles in the entorhinal cortex - A small number of senile plaques in the temporal cortex and almost none in the hippocampal formation. |
| **PDD #4** | F | 84 | 72 | Unknown | - Tremulous Parkinsonism with hallucinations, dementia and mild dyskinesia. - LBD brain stem predominant, - Clinical PD | Entorhinal grade 1 and isocortical grade 3 Aβ /tau pathology | - A few LBs in the superior frontal gyrus - Many LBs in the amygdala, mesial temporal neocortex, Ammon’s horn, subiculum, entorhinal cortex and fusiform gyrus. - Deposition of α-synuclein prominent in the CA2 - Numerous LBs in SN | - Many plaques with tau in the superior frontal gyrus - P-tau deposition in amygdala, mesial temporal neocortex and SN - Numerous plaques in the temporal neocortex, subiculum, entorhinal cortex and CA3-CA1. - Strong tau in CA4-CA1 - Tau prominent in the CA2 |
| **PDD #5** | M | 80 | 60 | Unknown | - Parkinsonism progressing with typical course and cognitive decline - LBD, brain stem predominant. | Entorhinal grade 2 and isocortical grade 1 Aβ /tau pathology | - LBs in the frontal and entorhinal cortex - Pre-synaptic terminals in the frontal cortex, hippocampus, and entorhinal cortex. - SN, nucleus basalis, amygdala, and locus coeruleus are all severely affected - A few LBs in the red nucleus - Severe accumulation of α-synuclein in raphe nuclei, reticular formation and motor nucleus of vagus. | - No p-tau accumulation but has Aβ plaques in the superior frontal gyrus - P-tau in hippocampus and subiculum and entorhinal cortex - Tau and Aβ plaques in CA1 and subiculum - A few tau in midbrain |
| **PDD #6** | M | 83 | 73 | Unknown | - Parkinsonism with cognitive & autonomic involvement - LBD, brain stem predominant. | N.A. | - No significant α-synuclein pathology in the superior frontal gyrus - The hippocampus, the subiculum and the entorhinal cortex show a few LBs - Alpha-synuclein in the nucleus basalis and moderate in the amygdala and adjacent temporal cortex - LBs in the substantia nigra and midbrain | - No p-tau and Aβ accumulation in the superior frontal - Significant p-tau accumulation in CA1-2 sectors and subiculum - The CA3-4 and the entorhinal cortex are less severely involved. No accumulation of Aβ. |
| **PDD #7** | F | 78 | 59 | Chest infection and late stage PD | - Tremulous Parkinsonism, autonomic and neuropsychiatric involvement - LBD, brain stem predominant | Isocortical grade 0 and entorhinal grade I Alzheimer-type pathology | - The superior frontal gyrus demonstrates diffuse granular immunolabelling and a few LBs - The nucleus basalis shows moderate involvement - Significant α-synuclein deposition in CA4-2, CA1 and subiculum are much less affected - SN moderately involved | - No tau or Aβ-related pathology in the section of frontal lobe - P-tau accumulation in entorhinal cortex in neuropil and cell bodies - A few tau-positive axons in Ammon’s horn with no Aβ deposits - No tau pathology in midbrain |
| **PDD #8** | F | 83 | 80 | Chest infection; stroke; PD | - Tremulous Parkinsonism with autonomic features & dementia (PDD) - LBD, brain stem predominant | Isocortical grade 1 entorhinal/hippocampal grade 0 Alzheimer’s type pathology | - Mild α-synuclein pathology in superior frontal gyrus, cingulate gyrus, prefrontal gyrus, CP, pallidus, hypothalamus, thalamus and amygdaloid complex - No deposits in temporal cortex, primary visual cortex, parietal lobule and cerebellum - Moderate in nucleus basalis, SN and locus coeruleus - Less prominent in hypothalamus, spinal trigeminal nucleus and reticular formation of the medulla. | - No tau pathology in superior frontal gyrus and hippocampus - Some plaques in frontal lobe and primary visual cortex and only a few diffuse plaques in the entorhinal cortex. - No tau deposition in midbrain. |
| **PDD #9** | M | 75 | 50 | Bronchopneumonia, PD, Senile dementia | - Tremulous Parkinsonism with autonomic & neuropsychiatric involvement (PDD) - LBD, neocortical type - Tau pathology | Braak Stage II | - Moderate LBs in superior frontal, temporal, parietal and visual cortices - LBs in the nucleus accumbens, CP - Many LBs and neurites in amygdala - Many α-synuclein in CA2 and entorhinal cortex in hippocampus - In the lower midbrain, SN shows many LBs, Lewy neurites and granular cytoplasmic staining. | - Senile plaques in the superior frontal gyrus and a few in hippocampus and occipital cortex - tau positive neuropil threads in CA2 and subiculum, other areas of hippocampus show occasional neurofibrillary tangle. |
| **PDD #10** | M | 81 | 71 | Unknown | - Tremulous parkinsonism with progression to dementia (PDD) - Imaging evidence of cerebrovascular disease - LBD, neocortical type. - Tau pathology | Braak Stage II | - A few LBs in the frontal, temporal, precentral, and parietal cortices and basal forebrain. - A few LBs in CP and septal nuclei - Alpha-synuclein deposits in the hypothalamus and thalamus at the level of the rostral substantia nigra - The amygdala is severely affected with LBs, Lewy neurites and granular cytoplasmic deposits - A few deposits in all subfields of the hippocampus, entorhinal cortex and fusiform gyrus. - LBs, neurites and granular cytoplasmic deposits in SN in midbrain | - A few Aβ plaques in basal forebrain - Occasional tau tangles in the superior frontal gyrus and basal forebrain and CP - A few neurofibrillary tangles in the CA2-1 of Ammon’s horn, more in the subiculum and entorhinal cortex - Occasional neuronal and neuritic deposits in SN, oculomotor nucleus and reticular formation in the section of the midbrain. |
| **DLB #1** | M | 58 | 43 | Acute myeloid leukaemia | - LBD, diffuse type. - Young onset Parkinsonism, progressing to dementia with hallucinations and autonomic involvement & dyskinesia. | Isocortical grade 1 and entorhinal/hippocampal grade 2 Alzheimer type pathology | - LBs in the lower cortical layers of the superior frontal gyrus and in the lower cortical layers of the entorhinal cortex and fusiform gyrus. - LBs in the amygdaloid nucleus and the adjacent temporal cortex - Lewy neurites are prominent in the CA2 region of the hippocampus. Extensive involvement of the nucleus basalis, the substantia nigra, the locus coeruleus, the dorsal motor nucleus of the vagus and the reticular formation. | - Diffuse and neuritic plaques in the superior frontal gyrus but no significant tau-related pathology - Aβ positive and tau-positive neuritic plaques in hippocampus - Diffuse tau accumulation in the neuropil of the entorhinal cortex - No tau deposits in the midbrain. |
| **DLB #2** | M | 70 | 69 | Bronchopneumonia | - DLB with rapidly progressive course and parkinsonian features. - LBD, brain stem predominant | Isocortical grade II and entorhinal/hippocampal grade II Alzheimer type pathology | - No α-synuclein in superior frontal gyrus - Hippocampus and amygdala are mildly affected - Moderate LBs in nucleus basalis, substantia nigra and locus coeruleus - Minimal in the reticular formation of the pons and medulla | - Several neuritic plaques in the superior frontal gyrus - Aβ mainly in intracortical and leptomeningeal vessels and very few neuritic plaques - P-tau in subiculum and entorhinal cortex in hippocampus - Neurofibrillary tangles in subicular cortex & plaques without tau in entorhinal cortex. |
| **DLB #3** | M | 63 | 54 | Bronchopneumonia | - LBD, brain stem predominant - Severe co-existent Alzheimer pathology - “Parkinson’s with LBs” with progression to severe autonomic & neuropsychiatric involvement | Isocortical grade 4 and hippocampal/entorhinal grade 3 Alzheimer’s type pathology | - Some LBs in all cortical areas - Microglial staining in visual cortex. - Mild to moderate involvement in nucleus accumbens, CP, globus pallidus and thalamus - Extensive involvement in nucleus basalis - Alpha-synuclein in the posterior hypothalamus and in the amygdaloid complex - Some LBs in CA3-4 and more extensive in CA2 of hippocampus - Intra- and extra-cellular LBs and neurite staining in the midbrain, the SN. | - Widespread focal and diffuse Aβ in frontal, temporal, entorhinal and primary visual cortices - Diffuse plaques in the CA1-2 of hippocampus - P-tau and plaque-associated neuritic clusters and neurofibrillary tangles in frontal and entorhinal cortices - Intense neurites in the CA2 sector. - A few tau-positive fibres in SN. |
| **DLB #4** | F | 83 | 80 | Unknown | - Tremulous Parkinsonism, micrographia & memory impairment - Prominent neuropsychiatric features. - LBD, brainstem predominant. | Isocortical grade 1 and hippocampal/entorhinal grade 3 Alzheimer’s type pathology | - Occasional cortical LBs in the frontal, paraolfactory, temporal, primary visual and motor cortices. - Mild involvement in accumbens, CP, globus pallidus, posterior hypothalamus, thalamus, and the accessory cortical nucleus of the amygdala - Moderate involvement in the nucleus basalis - Neuritic staining in the CA2 in the hippocampus - Mild changes with a few LBs and Lewy neurites in SN of the midbrain - No significant α-synuclein in the spinal cord | - Aβ plaques but no p-tau in the frontal and occipital cortices - Numerous diffuse Aβ deposits throughout the hippocampus, subiculum and entorhinal cortex. - Markable tau tangles in CA1-2, the subiculum, and entorhinal cortex - Occasional positive fibres in the SN and red nucleus of the midbrain. |
| **DLB #5** | M | 76 | 70 | Cerebrovascular Disease; Lewy Body Dementia | - DLB, limbic (transitional) type | Poor memory, Dementia, | - The neocortex and the hippocampus show varying degrees of LB pathology with the cingulate cortex being most significantly affected - Septal nuclei, CP, globus pallidus, hypothalamus and amygdaloid complex show sparse LB pathology - No deposits in thalamus and cerebellum. - The nucleus basalis of Meynert, the substantia nigra and coeruleus complex are mildly affected | - A few neurofibrillary tangles in Ammon’s horn, subiculum and entorhinal cortex but not in the frontal or occipital cortices - tau deposits in lateral ventricle - Moderate Aβ plaques in the frontal lobe, subiculum, entorhinal cortex and primary visual cortex - No deposits of tau in midbrain |
| **DLB #6** | F | 82 | 77 | Unknown | - LBD, neocortical type - Tremulous Parkinsonism with hallucinations & cognitive decline | Braak stage III Alzheimer-type | - Numerous LBs and glial in the frontal, cingulate, temporal, entorhinal and parietal cortices - The hippocampus exhibits intense neuritic staining in the CA2 - SN shows significant loss of pigmented neurons | - Aβ plaques throughout the frontal and temporal cortex, the striatum and the hippocampus - Occasional neurofibrillary tangles, and neuritic clusters in the frontal and temporal cortex |
| **DLB #7** | M | 78 | 72 | Unknown | - LBD, early neocortical-type - PDD vs. DLB Memory problems & Left arm tremor & stiffness | Memory problems | - Numerous LBs in the cingulate and entorhinal cortices, some in the superior frontal cortex and only a few in the occipital cortex - Neuritic in the CA2 in hippocampus - Severe to moderate LB and neurite pathology in SN, locus coeruleus, dorsal nucleus of the vagus and the intermediate reticular zone | - No Aβ in any of the sections stained - tau pathology is restricted to a few scattered neurons in entorhinal cortex, amygdala, subthalamic nucleus, hippocampus and brainstem. |
| **DLB #8** | F | 76 | 71 | Lewy body dementia | - DLB - LBD neocortical type - Alzheimer-type tau pathology | Braak stage V | - Numerous LBs associated with glial deposits in the superior frontal gyrus, basal forebrain, cingulate, insular and occipital cortices - Some granular α-synuclein in striatum - LBs and neurites in rostral SN in thalamus - Widespread deposits in the amygdaloid complex and overlying temporal cortex. - Severe pathology in the entorhinal cortex and fusiform gyrus. | - Several Aβ deposits and plaques in anterior frontal, basal forebrain, entorhinal, temporal and occipital cortices, and whole hippocampus - Widespread cored deposits in CP - Neurofibrillary tangles and neurites in frontal and temporal cortex - Occasional neurofibril tangles in CP and midbrain |
| **DLB #9** | M | 77 | 74 | Aspiration pneumonia and LBD | - LBD, neocortical type - Tau pathology - Aβ pathology in the striatum. - Akinetic-rigid syndrome | Braak stage II | - Occasional cortical-type LBs - Many LBs in the nucleus basalis of Meynert and in the hypothalamic ventromedial nucleus - Involvement of the rostral SN - Slight involvement of the substantia nigra, reticular formation and oculomotor nucleus - No deposits are present in the spinal cord. | - Plaques in the frontal gyrus, insula, entorhinal and occipital cortices - Many diffuse plaques in striatum - No tau pathology in anterior frontal and occipital cortices - Very occasional tangles in thalamus and midbrain. |

**AD =** Alzheimer’s disease**, ARTAG** = Aging-related tau astrogliopathy, **CA** = Cornu Ammonis, **CAA** = Cerebral amyloid angiopathy, **CERAD** = Consortium to Establish a Registry for Alzheimer’s Disease, **CP** = caudate and putamen, **DLB** = Dementia with Lewy body, **LB =** Lewy body**, LBD =** Lewy body disease, **NIA-AA** = National Institute on Aging–Alzheimer’s Association, **N.A.** = Not available, **PART** = Primary age-related tauopathy, **PD** = Parkinson’s disease, **PDD** = Parkinson’s disease dementia, **SN** = substantia nigra

**Supplementary Figures**

**Supplementary Fig. 1**

**
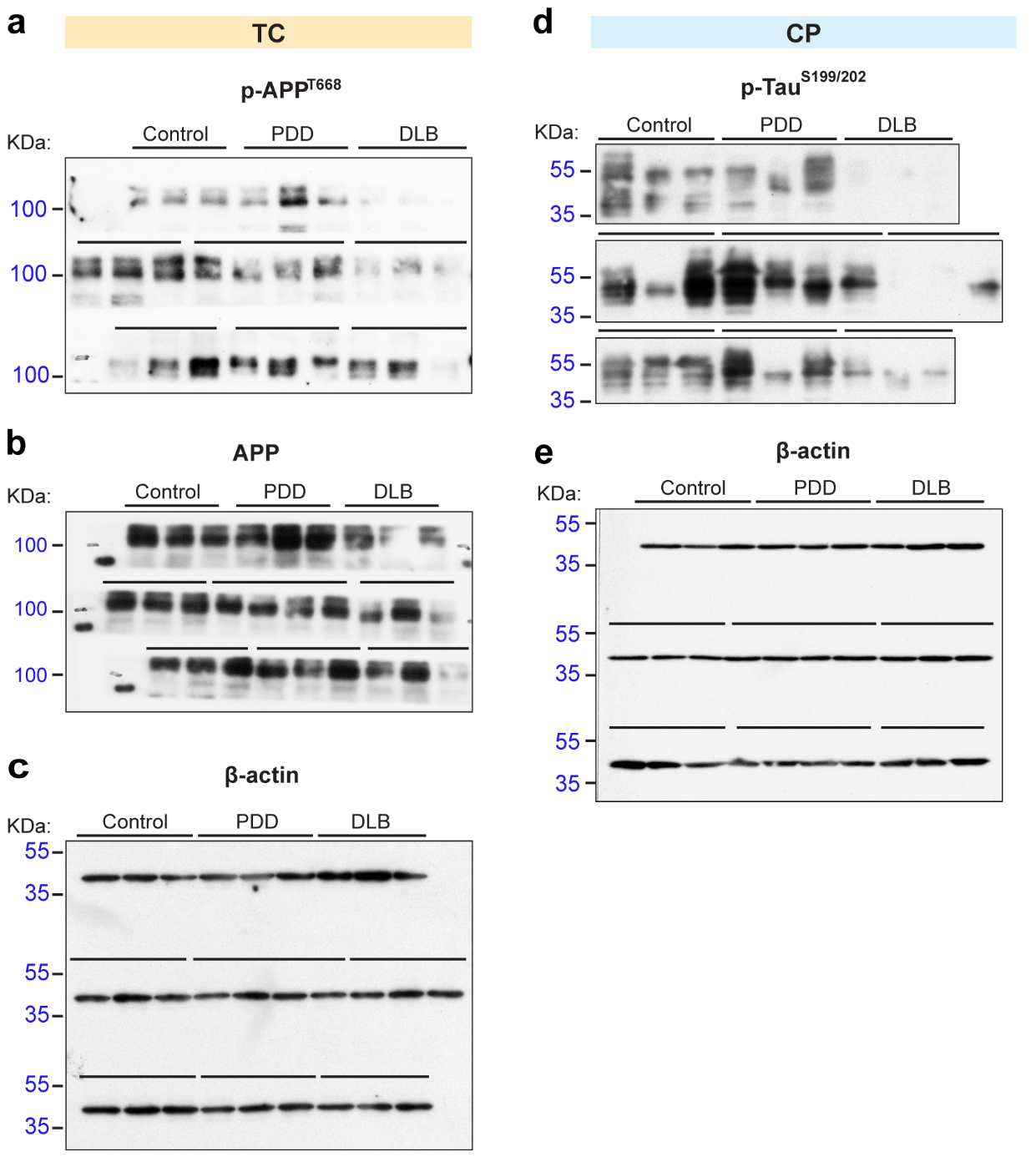
**

**Supplementary Fig. 1.** **AD-related biomarkers tested in the TC and CP regions of Control (Ctrl), PDD, and DLB post-mortem samples.** Multivariate analysis of post-mortem samples from the TC and CP regions of controls and individuals with PDD and DLB. Western blotting analyses were performed for AD-related biomarkers. Blots were cropped from 3 gels after SDS-PAGE before immunoblotting. (**a**) p-APP^T668^, (**b**) APP, and (**c**) β-actin in the TC region. (**d**) p-Tau^S199/202^ and (**b**) β-actin in the CP region.

**Supplementary Fig. 2**

**
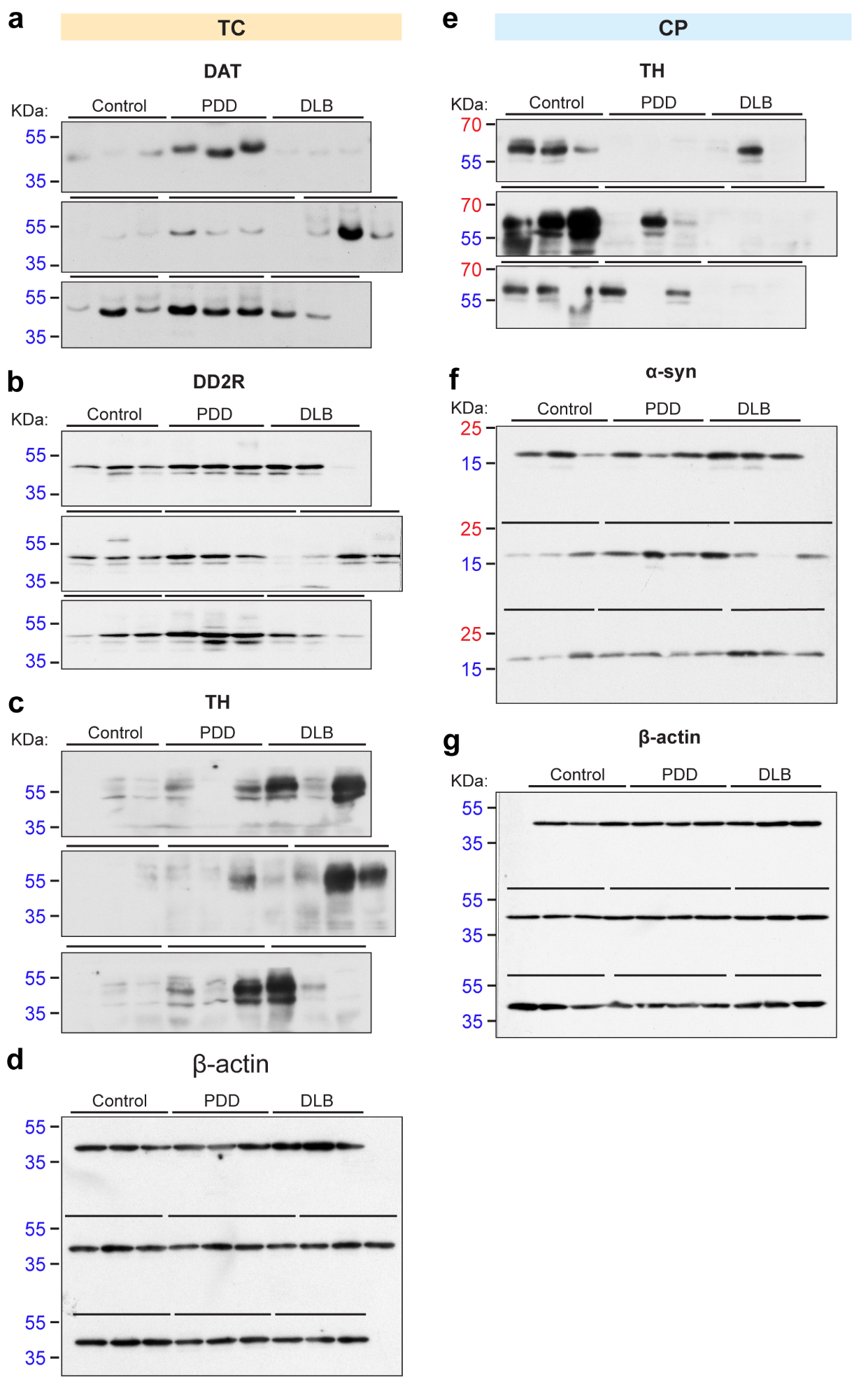
**

**Supplementary Fig. 2.** **PD-related biomarkers tested in the TC and CP regions of Control (Ctrl), PDD, and DLB post-mortem samples.** Multivariate analysis of post-mortem samples from the TC and CP regions of controls and individuals with PDD and DLB. Western blotting analyses were performed for PD-related biomarkers. Blots were cropped from 3 gels after SDS-PAGE before immunoblotting. (**a**) DAT, (**b**) DD2R, (**c**) TH, and (**d**) β-actin in the TC region. (**e**) TH, (**f**) α-syn, and (**g**) β-actin in the CP region.

**Supplementary Fig. 3**


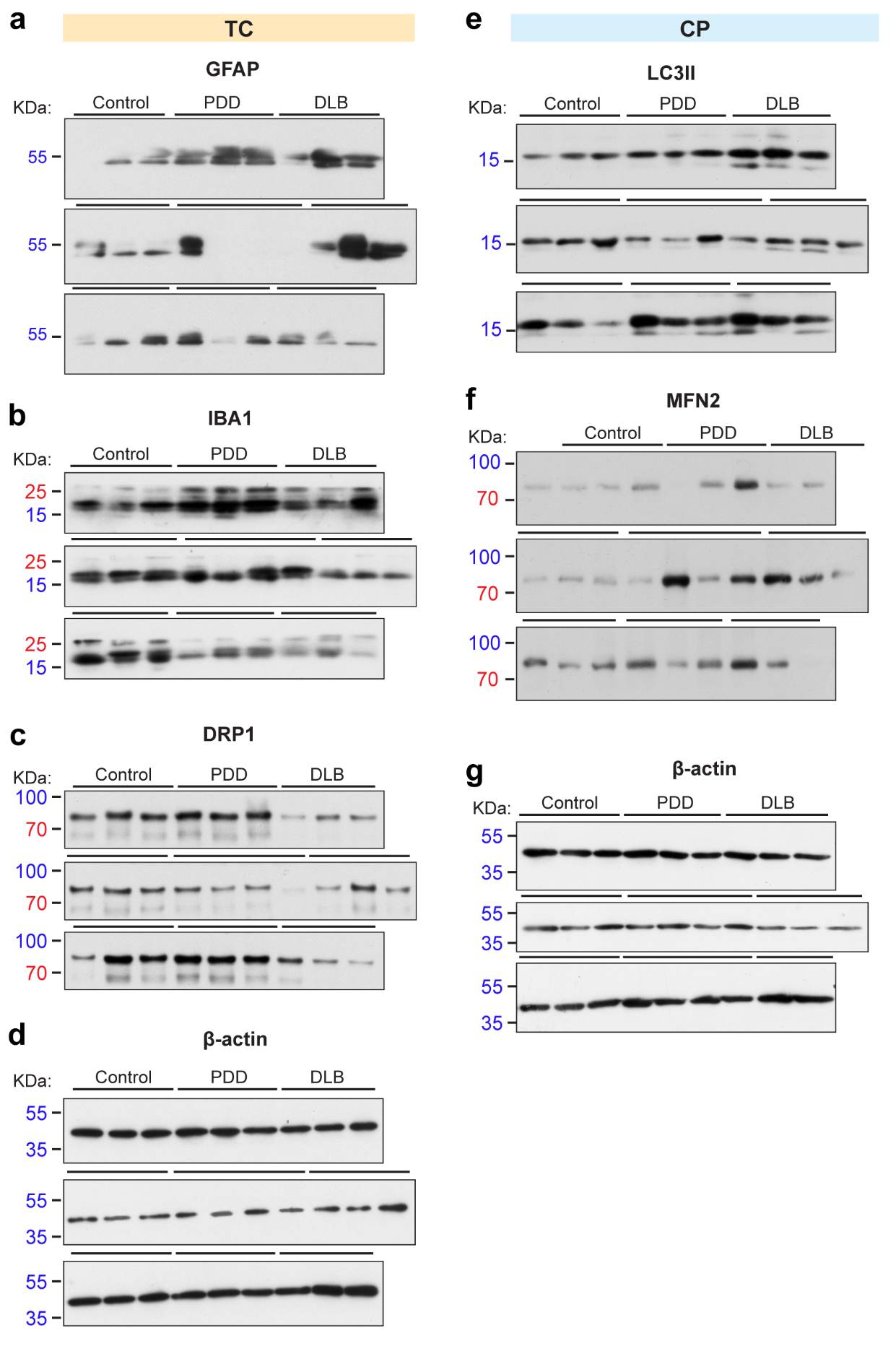


**Supplementary Fig. 3.** **Mitochondrial, neuroinflammation, and autophagy-related biomarkers tested in the TC and CP regions of Control (Ctrl), PDD, and DLB post-mortem samples.** Multivariate analysis of post-mortem samples from the TC and CP regions of controls and individuals with PDD and DLB. Western blotting analyses were performed for PD-related biomarkers. Blots were cropped from 3 gels after SDS-PAGE before immunoblotting. (**a**) GFAP, (**b**) IBA1, (**c**) DRP1, and (**d**) β-actin in the TC region. (**e**) LC3II, (**f**) MFN2, and (**g**) β-actin in the CP region.

**Supplementary Fig. 4**


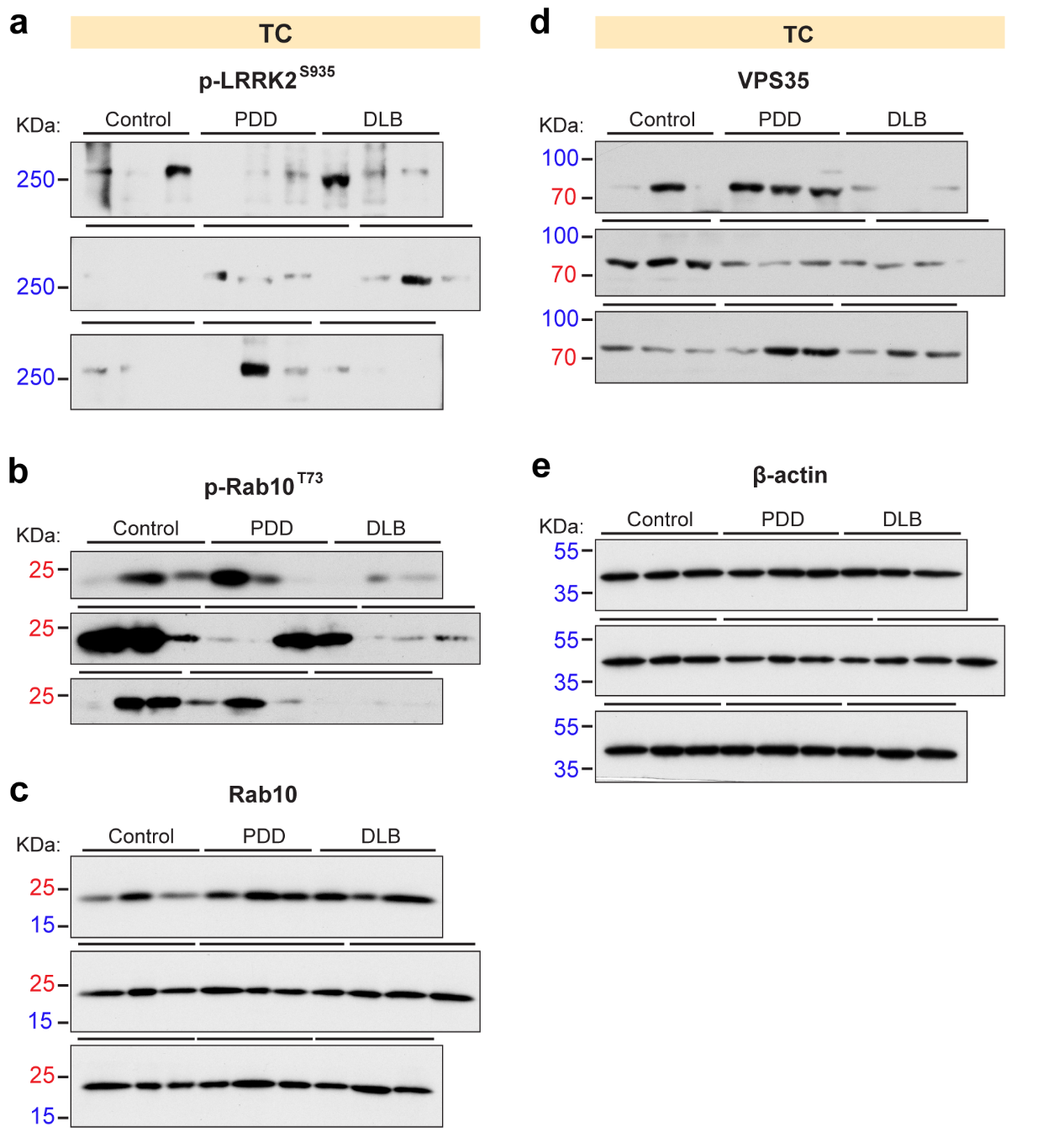


**Supplementary Fig. 4.** **LRRK2 substrate proteins tested in the TC region of Control (Ctrl), PDD, and DLB post-mortem samples.** Multivariate analysis of post-mortem samples from the TC and CP regions of controls and individuals with PDD and DLB. Western blotting analyses were performed for PD-related biomarkers. Blots were cropped from 3 gels after SDS-PAGE before immunoblotting. (**a**) p-LRRK2^S935^, (**b**) p-Rab10^T73^, (**c**) Rab10, (**d**) VPS35, and (**e**) β-actin in the TC region.
